# Switching of behavioral modes and their modulation by a geometrical cue in the ciliate *Stentor coeruleus*

**DOI:** 10.3389/fcell.2022.1021469

**Published:** 2022-11-01

**Authors:** Syun Echigoya, Katsuhiko Sato, Osamu Kishida, Toshiyuki Nakagaki, Yukinori Nishigami

**Affiliations:** ^1^ Graduate School of Life Science, Hokkaido University, Sapporo, Japan; ^2^ Research Institute for Electronic Science, Hokkaido University, Sapporo, Japan; ^3^ Field Science Center for Northern Biosphere, Tomakomai Experimental Forest, Hokkaido University, Tomakomai, Japan

**Keywords:** geometrical cue, ciliates, *Stentor*, spatial exploration, behavioral transition, protists

## Abstract

Protists ubiquitously live in nature and play key roles in the food web chain. Their habitats consist of various geometrical structures, such as porous media and rigid surfaces, affecting their motilities. A kind of protist, *Stentor coeruleus,* exhibits free swimming and adhering for feeding. Under environmental and culture conditions, these organisms are often found in sediments with complex geometries. The determination of anchoring location is essential for their lives. However, the factors that induce the behavioral transition from swimming to adhering are still unknown. In this study, we quantitatively characterized the behavioral transitions in *S. coeruleus* and observed the behavior in a chamber with dead ends made by a simple structure mimicking the environmental structures. As a result, the cell adheres and feeds in narrow spaces between the structure and the chamber wall. It may be reasonable for the organism to hide itself from predators and capture prey in these spaces. The behavioral strategy for the exploration and exploitation of spaces with a wide variety of geometries in their habitats is discussed.

## 1 Introduction

Animal navigation is widely observed in nature. For example, birds perform homing behaviors (Landreth and Ferguson, 1967) that have been actively studied in neuroscience (Capaldi et al., 1999), and ants explore the locations of food, which can be explained by simple algorithms such as cellular automata (Deneubourg et al., 1990). Even in bacteria and protists, a navigation ability is required to survive, and their habitats consist of diverse microscopic structures that affect their behaviors. Indeed, the behavioral responses of these organisms have been studied in various microspace geometries: porous media (Bhattacharjee and Datta, 2019), rigid surfaces (Kantsler et al., 2013; Ohmura et al., 2018, 2021), dead ends (Kunita et al., 2014), corners (Théry et al., 2021), interstices between inclined plates (Ishikawa and Kikuchi, 2018) and confined areas (Beppu et al., 2017, 2021; Ostapenko et al., 2018; Bentley et al., 2021). From these studies, it was determined that cell behaviors change in response to the geometrical structures of living spaces.

A kind of protist, namely, ciliates, live in various natural environments, such as fresh water and even the deep sea (Hausmann et al., 2002; Lynn, 2017). These organisms play key roles in the food web chain in the hydrosphere (Sanders and Wickham, 1993; Lynn, 2008). Many kinds of ciliates *Vorticella* (Sleigh and Barlow, 1976), *Opercularia* (Sládecek, 1981; Hartmann et al., 2007) and *Stentor* (Tartar, 1961) anchor to underwater surfaces and consume bacteria, small ciliates and small detritus. Alternatively, they are preyed upon by large multicellular organisms. Thus, their behaviors play an important role in ecosystems in their habitats (Sherr and Sherr, 2002).

*Stentor* is a large single-celled organism (0.5–1 mm) belonging to the phylum Ciliophora in the order Heterotrichida (Adl et al., 2019); this organism has been well studied for wound healing and regeneration (Tartar, 1961; Blauch et al., 2017; Marshall, 2021; Zhang et al., 2021). The entire body surface of *Stentor* is covered in cilia for swimming. On the edge of the oral region (peristome), the cilia are also tightly packed (membranelle), i.e., two or three rows of cilia (20–25 cilia in each row (Randall and Jackson, 1958)). The membranelles are components of a ring-like oral structure (membranellar band). The membranelles collectively beat and stir the fluid to generate a feeding vortex (Maupas, 1888), which brings food to the oral apparatus.

*Stentor* exhibits free swimming. They become sessile once attached to the structures such as dead leaves and twig duckweed, *Spirogyra* mats and dead cattail leaves (Tartar, 1961). Switching its motility may depend on the intra- and extracellular conditions. Light affects their behaviors; that is, a negative photoresponse can be caused by the colored pigment stentorin under the near cell surface (Tartar, 1961; Song et al., 1980a, 1980b; Iwatsuki and Song, 1989; Iwatsuki, 1992). Recently, Trinh et al. reported that “complex decision-making” in *Stentor* (Dexter et al., 2019) relates to intracellular conditions (Trinh et al., 2019).

In their behavior, *Stentor* mainly exhibits three shape states: a droplet, a cone and a trumpet (Tartar, 1961). In free swimming, the cell forms a cone shape; on the other hand, trumpet-shaped cells slowly swim or attach to substances for feeding. Mechanical and electrical stimuli produce a quick contraction from cone or trumpet shapes to droplet shapes as an avoidance behavior. During the adherence process, the *Stentor* anchors to a surface using an adherent structure (holdfast) at the posterior end, in which mucus is secreted (Andrews, 1945). The determination of anchoring location is essential for capturing prey and hiding from predators. However, it is still unclear what external cues induce the behavioral change from swimming to adhering. In this study, we quantitatively evaluated the effect of extracellular structures on *Stentor*’s behavior.

## 2 Materials and methods

### 2.1 Cell cultures

*S. coeruleus* was collected from the pond at Tomakomai located Hokkaido, Japan (42.67°N, 141.59°W) and was maintained in a 96-well cell culture plate (TR5003, TrueLine, United States; 6.4 mm diameter) containing filtered pond water using a Millex 0.22 µm fileter (Merck Millipore, Burlington, MA, United States) at room temperature in the dark. We placed *Cryptomonas paramecium* into the culture plate as the *Stentor*’s feed, which was axenically grown in a CHM medium [0.1% CH_3_COONa·3H_2_O, 0.1% “Lab-Lemco” powder (Oxoid L29) in Milli-Q water; The pH 6.9 ± 0.1 with 1 N NaOH] was adjusted under light-dark conditions (12 h:12 h) at room temperature every three or 4 days. We transferred *C. paramecium* to a fresh CHM medium every week.

In the experiments using chambers with/without structure, cells were collected from the Shiribetsu River located in Hokkaido, Japan (42.81°N, 140.69°W) and cultured at 25°C in a 6 cm diameter plastic dish containing modified Peters’ solution (0.55 mM CaCl_2_, 0.15 mM MgSO_4_, 0.15 mM K_2_CO_3_ and 0.75 mM Na_2_CO_3_, adjusted pH 7.4 ± 0.1 with 1 N HCl) in the dark (de Terra, 1966) with one wheat grain (MRP-706, Marukan, Osaka, Japan). The cells were transferred into a fresh medium to maintain the cultures every 2–3 weeks. The difference in the strain was not significant in our experiment (detailed in Supplementary Figure S1).

### 2.2 Observation of the shape transitions of the cell

Before the observations, we washed the cells in fresh pond water twice and starved them for 3 days. Prior to transferring a cell into an observation chamber, we washed the cell with fresh pond water again and equilibrated it for over 20 min. The observation chamber was made of a silicon sheet (244-6012-03, HAGITEC Co., Ltd., Chiba, Japan; 0.2 mm depth, 5 mm inner diameter) and covered by a cover slip (Supplementary Figure S2). Cell behavior was observed by an inverted microscope IX73 (Olympus, Tokyo, Japan) equipped with a PLANPO X1.25 objective (Olympus, Tokyo, Japan). Images were recorded by using a CMOS camera (ORCA-Flash4.0 v2, Hamamatsu Photonics, Hamamatsu, Japan) with an exposure time of 5.0 ms and a frame rate of 10 frames per second (fps) under a brightness field using weak 700 nm light (M700L4, Thorlabs, New Jersey, United States) to reduce the photoresponse effects (Song et al., 1980a, 1980b; Iwatsuki, 1992; Song, 1999).

We used 14 cells to measure the swimming speed and the cell length over 25 min after placing them in the chamber. We recorded each cell for 24.7 ± 3.4 min (SD, 14 cells, maximum 32.0 min, minimum 18.6 min, total 345.1 min: Supplementary Figure S3). To measure the duration time of each shape transition and the diameter of the swimming trajectory in the trumpet shape, we recorded the cell behaviors and analyzed the data (Supplementary Figure S4). Because of the very low frequency at the droplet state, we recorded the behaviors of the cells in the droplet state by adding an external mechanical stimulus to obtain a transition vector and conducted computational classification of the cell state (Supplementary Figure S4).

Due to the speed of the contraction from the trumpet shape to the droplet shape, we recorded the contractions of eight cells by the high-speed camera FASTCAM Mini AX50 (Photron, Tokyo, Japan) at 2,000 fps and an exposure time of 1/2,000 s. Images were collected by using the IX73 microscope with a UNLPlan X10 (Olympus, Tokyo, Japan) objective under 700 nm light.

When we observed the shape transitions from the trumpet shape to the droplet shape, we added an external stimulus by striking the chamber using a tweezer (Meister 0-SA, Rubis, Switzerland).

### 2.3 Image analyses and measurements of the center of the cell, swimming speed, cell length and swimming trajectory

First, we analyzed the data containing no external stimulus. We cutoff sequential images to several sections (2,000–5,000 frames) and obtained each background image by calculating the local median intensity. Then, the sequential images subtracted from each background were subjected to Gaussian blurring (radius = 1.1 pixels), and binary images were acquired using the MaxEntropy threshold algorithm (Kapur et al., 1985). After that, we obtained the centers *C*_*i*_: ***r***_*i*_ = (*x*_*i*_,*y*_*i*_) (mm) of the cells at the *i*th frame. To measure the cell length, we obtained the length *l*_*i*_ (*θ*) from the center of the cell to the edge by converting images to polar coordinates by 2° each (Supplementary Figure S5). The cell length *L*_*i*_ was defined by *L*_*i*_ = *l*_*i*_ (*θ*_1_) + *l*_*i*_ (*θ*_2_), where *l*_*i*_ (*θ*_1_) and *l*_*i*_ (*θ*_2_) are the top two local maxima of *l*_*i*_ (*θ*), and *θ*_1_ and *θ*_2_ denote the directions of the anterior and posterior directions, respectively (Supplementary Figure S5).

Instantaneous swimming speeds *s*_*i*_ = | ***v***_*i*_| were calculated from the center of the cell *C*_*i*_, defined by ***v***_*i*_ = 0.5 [(***r***_*i+*1_−***r***_*i-*1_) · fps] (mm/s). Because of the difficulties of obtaining the shape and the center of the cell due to the thin posterior region and the fusing to the air bubble, we obtained a smoothed time series of the cell length *L*’_*i*_ and speed *s*’_*i*_ from the median values for ± 5 s (50 frames) for displaying the overview of behaviors; ± 3 s (30 frames) for obtaining the time duration from cone to trumpet; and ± 1 s (10 frames) for obtaining the time duration from droplet to cone and from droplet to trumpet.

In the contraction process from trumpet to droplet by adding an external mechanical stimulus, we manually extracted three points of the posterior end (P) and two edges of the oral apparatus, OA_1_ and OA_2,_ due to defocusing in striking. Then, we calculated the middle point (M) between OA_1_ and OA_2_. After that, the cell length *L* was determined as the distance of the MP.

### 2.4 The estimation of the frequencies of each cell state

To obtain an overview of the behaviors and compare them with the computational classification of the cell states (see Section 2.6), we divided the behaviors into three states (droplet, cone and trumpet) with reference to Tartar’s descriptive classification (Tartar, 1961). Then, we obtained the frequency of the cell states and the distributions of the cell length and swimming speed at each state. The normalized length $\tilde{L}$ and speed $\tilde{s}$ were obtained by calculating the averaged length *L*_0_ and speed *s*_0_ in each free-swimming cell in the cone state for 10 s (the parameters were the same in all experiments). The normalized values at the *i*th frame defined by $\tilde{L}$ _*i*_ = *L*’_*i*_/*L*_0_, $\tilde{s}$ _*i*_ = *s*’_*i*_/*s*_0,_ where *L*’_*i*_ and *s*’_*i*_ were the median values for ± 5 s (the parameter was the same in all experiments) with regard to the length *L* and speed *s*, respectively. Exceptionally, the average length and speed in 1 sample in the gel chamber with a structure were determined to be *L*_0_ = 0.5 and *s*_0_ = 0.9 because most of the state in this cell was adhesion, and we could not extract averaged values at the swimming state. The bin width was 0.03 (normalized).

All image analyses were conducted by using Python 3.7.1 (https://www.python.org/) and ImageJ/Fiji (Schindelin et al., 2012).

### 2.5 Obtaining the vector field of the behavioral transition of the cell

We described the behaviors of cells in a 2D state field. The field consists of the normalized length $\tilde{L}$ and speed $\tilde{s}$ (averaged values were calculated from the data for ± 10 s). A dot ***S***_*i*_: ( $\tilde{L}$ _*i*_, $\tilde{s}$ _*i*_) and a trajectory connecting those dots in the field represent the cell state at frame *i* and the sequential behavior of a cell, respectively. To obtain these data, we used ± 5 s median values. When a cell remained in the same state, these dots and trajectories accumulated in a region. To derive the directions of the trajectories, we defined the transition vector field of behavioral transition ***v***_*i*_, defined by ***v***_*i*_ = ***S***_*i*+1_−***S***_*i*−1_. To obtain smoothed vectors, we calculated the averaged vector in ± 5 s.

Then, we derived the vector field of the transition of the cell state, and we divided the state field into a grid pattern at regular interval 0.1 (normalized). We calculated the median vector in a grid. We eliminated the vector in the grid less than 0.05%.

### 2.6 The computational classification of cellular states in the state field

We eliminated the transition periods (±15 s of droplet state, ±60 s of cone state, ±30 s of trumpet state) to classify the steady states of the cells computationally. Hierarchical cluster analysis was carried out to divide the cell states into three clusters using the “AgglomerativeClustering” function included in the “sklearn.cluster” library in Python (Pedregosa et al., 2011). The parameters used in the clustering method were the number of clusters (“n_clusters_”): 3, “affinity”: Euclidean, “linkage”: complete. Downsampling was applied to the data points (±5 s median values) by 10-increment sampling due to the limitation of the computational memory.

### 2.7 Measurements of the swimming trajectory and evaluation of the diameter of the local swimming trajectory in the trumpet shape

The swimming trajectories were obtained from the centers of the cell *C*_*i*_. To compare the trajectories of a cell in the cone state (swimming) and in the trumpet state (rotating), we extracted 400 frames (40 s) of sequential images from each swimming state (20 sections, 14 cells) and sequential images from each trumpet state (2502 ± 901 frames (SD, 20 sections); maximum 4435 frames; minimum 1111 frames; 14 cells).

The evaluation of the diameter of the local swimming trajectory in trumpet shape was conducted from each lap trajectory extracted from the trajectory in the rotating state (133 sections, 11 cells). The diameters of each trajectory were calculated by the maximum distance of the position (Supplementary Figure S6).

### 2.8 Estimations of the characteristic time of the shape transitions

From the obtained time series of cell length *L*_*i*_, we estimated the characteristic time of duration for the shape transitions. First, we obtained a smoothed time series of cell length *L’*_*i*_ from the median (following the above). The starting points of each shape transition were decided at the cross point of the line before the transition and after the transition (from cone to trumpet), the point of the minimum length from the contraction to after 20 s (from droplet to cone or trumpet) and the point at which external stimuli were added (from trumpet to droplet).

The duration times of each cell deformation were estimated from the deformation rate var *ε*(*t*) by fitting the following exponential functions. The deformation rate was defined as *ε*(*t*) = [*L*(*t*)−*L*_0_]/*L*_0_, where *L*_0_ is the cell length at the starting time of shape transitions in elongation or at the final state in contraction (*t* = 40 ms). In elongation, the rate was determined by calculating the averaged length for 10 s before each transition. To determine the duration times of each transition *τ*, the least-squares method (in Python using the package scipy. optimize.curve_fit) was conducted using the following function*:*

ɛ(t)=A·(1−exp(−t/τ))

In the contraction process, we used the mechanical model of the damped harmonic oscillator to model the contraction process in *Vorticella convallaria* (Moriyama et al., 1998). The model included the viscous resistance around the cell. The least-squares method was conducted by using the following function:

$$ɛ\left(t\right)=B_{1}\cdot \left(\exp\left(-t/\tau_{1}\right)\right){+B}_{2}\cdot \left(\exp\left(-t/\tau_{2}\right)\right).$$

The standard deviation of the time series of the strain was calculated for ±1 s in the elongations or ±0.5 ms in the contraction.

In the derivation of the correlation of the cell length and swimming speed, we calculated the normalized values by the maximum value of the speed in the transition is 1, and we defined the length at that time as 1. We fitted these values by power laws in two sections (1–1.25 and 1.25–1.45 about normalized length).

### 2.9 Preparation of the chambers made by agar to compare of two shapes

The observation chambers were obtained by mold casting in two processes: the preparation of prime molds made by polydimethylsiloxane (PDMS) and the formation of a gel chamber by pouring the agarose gel into PDMS prime molds. We made two kinds of chambers. One is the chamber (diameter 5 mm, depth 0.3 mm) with a disk-like structure (diameter 2.5 mm), and the another is the chamber (diameter 5 mm, depth 0.3 mm ) without any structure (Supplementary Figure S7).

The prime mold data were designed by Fusion 360 software (Autodesk, California, United States). The data were sent to a CAMM-3 Model PNC-3200 milling machine (Roland DG Corporation, Hamamatsu, Japan) by MODELA Player Version 3.7 (Roland DG Corporation, Hamamatsu, Japan), and prime molds were made by cutting a ZW-100 (Roland DG Corporation, Hamamatsu, Japan). Cutting involved three processes: surface cutting, rough cutting and finish cutting. Each cutting parameter is shown in Supplementary Table S1. The parameters in surface cutting and rough cutting were the same. For rough cutting and finish cutting of the chamber with structure, we used the micro end mill, MHRH230 φ0.2 × 2 (NS TOOL, Tokyo, Japan). In other processes, the end mill 2CEM-G-2.5D-2 (MonotaRO, Hyogo Japan) was used.

PDMS was prepared by mixing the prepolymer and the curing agent of the SYLGARD™184 silicone elastomer kit (Dow Corporate, Michigan, United States) in a 10:1 (weight:weight) ratio. The mixture was mixed for 20–30 min by plastic stick by hand. After that, it was vacuum-degassed for approximately 1 h. The mixture was poured into molds and vacuum-degassed again for approximately 1 h. Then, PDMS prime chambers covering a glass slide were horizontally kept at 25°C. After 48 h, the PDMS prime chambers were removed from the molds.

Prior to obtaining the observation chambers, the surface of the PDMS chamber was washed with ethanol and Milli-Q water and sonicated in reverse osmosis water. These processes were repeated three times or more. Then, the surface was rinsed with Milli-Q water. After drying, the surface was coated with plasma (PC-400T, STREX, Osaka, Japan) for 10–20 s for hydrophilization.

The mixture of the 2% (weight per volume) agarose (PrimeGel™ Agarose LE 1–20K, Takara Bio, Shiga, Japan) medium that melted the fresh modified Peters’ solution was poured into PDMS molds and covered by a cover slip to flatten the agar chambers. After 2–3 min, the cover slip was vertically removed, and the agar chambers were withdrawn from the PDMS prime mold. If we could not remove the agar chambers by the above methods, then the chamber was removed by sonication using the ultrasonic cleaner US-5KS (SND, Nagano, Japan) containing reverse osmosis water.

### 2.10 The observation method in the chamber with/without a structure

Prior to the observations, we washed the cells in a fresh medium twice and starved them for several hours. Then, we placed the gel chambers on a cover slip, and the surface of the chambers was wetted by the medium and covered on a glass slip with a plasma coating (20 s). After that, a glass slide on the chamber was slid until a swimming arena appeared, and the cell was transferred into the chamber from the edge of the glass slide by using a pipette tip. Its inside was coated with the 2% 2-methacryloyloxyethyl phosphorylcholine (MPC) polymer Lipidure^®^ CM5206 (NOF Corporation, Tokyo, Japan) in ethanol (weight per volume) to prevent the cell surface from adhering to the inside of a tip. In this operation, most of the cell quickly contracted due to external stimuli or hydrophilicity of the gel chambers; however, the cell swam after several seconds. Last, we slowly slid the cover slip to cover the observation chamber.

The behaviors were observed by using a stereomicroscope SZX16 (Olympus, Tokyo, Japan) equipped with a C-Mount Adaptor 0.5X, U-TV0.5XC-3 (Olympus, Tokyo, Japan) and a CMOS camera, ORCA-spark C11440-36U (Hamamatsu Photonics, Hamamatsu, Japan). The exposure time was 50 ms at 4 fps. A 2 × 2 binning scheme under a dim white brightness field, filtered by a red right pass plate for approximately 40 min, was utilized. The quantum sensor MQ-610 (Apogee Instruments, Utah, United States) could not detect the observation light due to weakness [<1 μmol s^−1^ m^−2^ ∼ 0.2 W/m^2^ (650 nm)]. Less than 0.2 W/m^2^ red light has little influence on the phototactic behavior (Song et al., 1980a).

### 2.11 The analyses of behavioral changes in the chamber with/without a structure

We obtained the behavioral data (center of the cell, swimming speed and cell length) according to Section 2.3. The different points were the obtained background images (using sequential images for 15 min by calculating local maximum intensity), the parameter of the Gaussian blur [radius = 0.5 (pixels)] and the threshold algorithm [triangle methods (Zack et al., 1977)]. When we could not acquire the exact cell position automatically, we manually excluded the noise and dust and prepared the threshold of the circularity or 4 pixel opening process (due to the high circularity of the cell compared with dust). After that, we obtained the position by the automatic method according to Section 2.3. To estimate the cell length, we converted the polar coordinate by 1° each. These acquired time series of length and speed are shown in Supplementary Figures S8, S9.

To calculate the local staying frequency in chambers, we divided the quasi-2D physical fields by 36 mm^2^ (6 mm × 6 mm) at regular intervals of 56 × 56 grids. Then, the probability density *p*(*x*_*i*_ *, y*_*j*_) in each grid was obtained from the number of observed centers of the cells in each grid. *p*(*x*_*i*_ *, y*_*j*_) was normalized as

$$\iint_{-3}^{3}p\left(x,y\right)dxdy=1$$

Adhesive spots of the cells, duration times and the directions of anterior end adherence were manually extracted from the sequential images. Crescent areas are defined as the region within 1 mm from the tip of the crescent. We did not count the cells staying for less than 10 s as adhesion. All statistical analyses were conducted in Python 3.7.1 using the “scipy.stat” package version 1.1.0.

## 3 Results

### 3.1 Cell behaviors in free swimming

**FIGURE 1:**
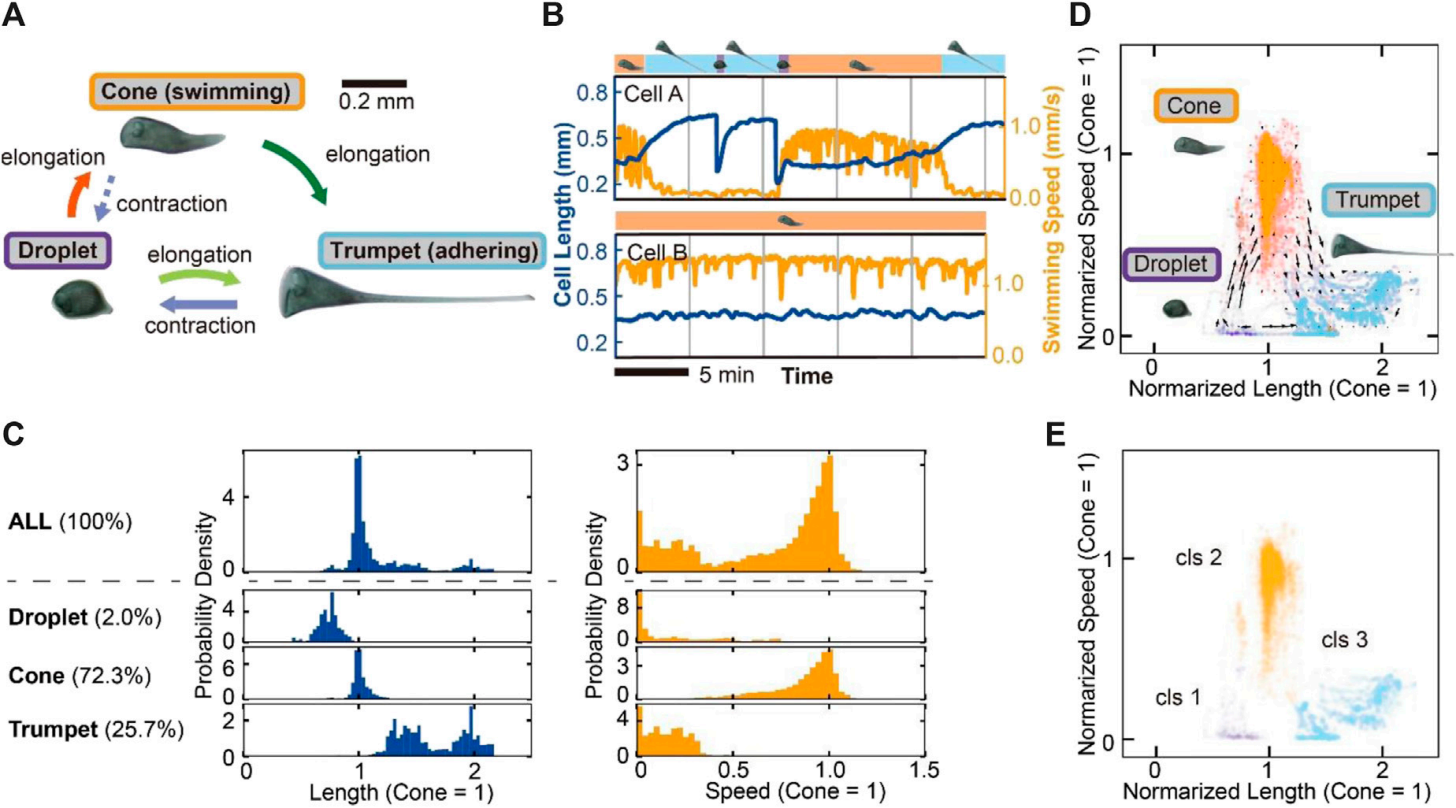
Three modes and transitions of *S. coeruleus* and the variety of behavioral modes in the quasi-2D disk chamber. **(A)** Schematic images of the cell states and the transitions. The cell takes three shapes (droplet, cone and trumpet). The cell elongates or contracts between these states, corresponding to each arrow. In the shape transition from cone to trumpet, the switching behavior occurs from swimming to adhering to a substrate. **(B)** The time series of cell length (blue line) and swimming speed (orange line). The graph displays the behaviors of two cells (Cell A, B). The cell states are shown on the top of each graph. Cell A often experiences a shape transition; on the other hand, Cell B remains in a swimming cone state for a longer observation time. **(C)** The frequency of the cell state and the distributions of the cell length and speed in each state. Each state was manually divided (droplet, 2.0%; cone, 72.3%; trumpet, 25.7%). The distributions of the length and the speed were normalized by calculating averaged values in the cone state. The length in the droplet is shorter than that in the cone. On the other hand, the length in the trumpet shape is higher than that in the cone. Swimming speed is separated into two regions, the swimming state (cone) and the adhering or lower swimming state (droplet and trumpet). **(D)** The transition vector of the cells. The dots represent the cell states in the 2D field containing the cell length and speed at time points. The speed and length are normalized by cone = 1. The dots were manually classified into three by cell state Tartar’s classification (droplet, purple; cone, orange; trumpet, light blue). **(E)** Hierarchical clustering of the steady cell states. The dots in the 2D state field are divided into three clusters. The computational clustering is similar to manual classification **(D)**.

After placing them in the chamber, we recorded the behavior of the cells in a quasi-2D disk chamber to perform quantitative measurements of the swimming speed and the cell length over 25 min. The cell exhibits three kinds of shape and behavioral transitions between these states (droplet, cone and trumpet) (Figure 1A). These shapes and transitions are consistent with the previous description (Tartar, 1961). In our experiments, some cells change their states frequently (Figure 1B, Cell A), while others take the steady cone state (Figure 1B, Cell B). There is a variation in the frequency of changing the states among those cells in this situation (Supplementary Figure S3). In the following analyses, we used the behavioral data containing these states. In our observation, cone-shaped cells and trumpet-shaped cells are observed in 72.3% and 25.7% of cells, respectively. Droplet-shaped cells are rarely observed in 2.0% (Figure 1C, Supplementary Figures S13A,B). The distributions of normalized length in each state (cone = 1) are distributed to three regions (Figure 1C). Cone-shaped cells perform free swimming, and droplets and trumpet-shaped cells mostly adhere or swim slowly. Then, we plotted the behavioral data in Figure 1C on the 2D state field. Figure 1D shows that the plots mainly form three clusters corresponding to each state. The vectors in the 2D field represent the velocity of the behavioral change. The graph allows the cell states and transitions in Figure 1A to be evaluated quantitatively and describes the behavioral flows between the cell states. Because two transitions from droplet to trumpet and from trumpet to droplet go through the same path toward mutually opposite directions in the state field, the velocities with respect to the behavioral changes are canceled and underestimated. Hierarchal clustering analysis, which connects the nearest clusters or dots repeatedly and makes several clusters, is conducted on the dots in the steady cell states (Figure 1E). When the computational clustering divides the cell state into three, the result corresponds to the classification in manual clustering (Figures 1D,E).

### 3.2 Shape transitions of the cells

**FIGURE 2:**
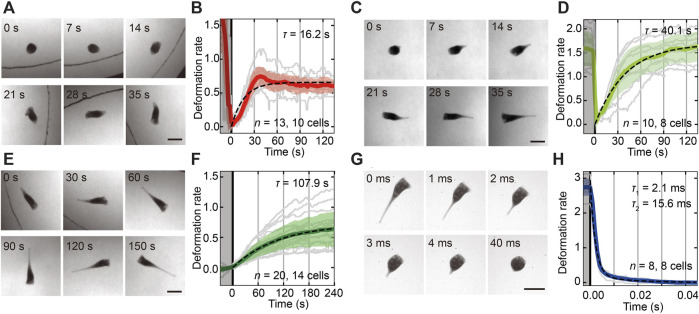
The shape transitions between the cell states and the estimation of each duration time of the transitions. **(A,C,E,G)** Sequential images of the shape transitions from droplet to cone **(A)**, from droplet to trumpet **(C)**, from cone to trumpet **(E)** and from trumpet to droplet **(G)**. The time is shown at the upper left in each image. The beginning time of the shape transition is adjusted at 0 s. In the transitions from droplet to cone **(A)** and cone to trumpet **(E),** the cell moves actively. The black curved line in **(A)** and **(E)** represents the boundary of the chamber. All bars are 0.2 mm. **(B,D,F,H)** The time series of shape deformation rates in each transition from droplet to cone **(B)**, from droplet to trumpet **(D)**, from cone to trumpet **(F)** and from trumpet to droplet **(H)**. The gray regions represent the sections before each transition. The colored solid lines are a time series of the averaged deformation rate. Individual data and the standard deviation of the rate are shown by gray lines and transparent colored regions, respectively. The duration times are estimated by exponential fitting to the averaged deformation rate represented as black dashed lines. These characteristic times are 16.2 ± 0.2 s (SD, *n* = 13, 10 cells) from droplet to cone **(A,B)**, 40.1 ± 0.51 s (SD, *n* = 10, eight cells) from droplet to trumpet **(C,D)**, and 107.9 ± 1.47 s (SD, *n* = 20, 14 cells) from cone to trumpet **(E,F).** The duration time from trumpet to droplet **(G,H)** is estimated at 2.1 ± 0.51 ms and 15.6 ± 2.3 ms (SD, *n* = 8, eight cells).

To characterize the behavioral transitions accompanied by the shape deformations in Figures 1A,D, we measured the time series of the cell length and estimated the duration times in each shape deformation (Figure 2). The duration time of each transition was estimated by exponential fitting to the averaged time series of the deformation rate. Because the contraction process from cone to droplet was observed only three times in total recording time (over 6 h), the process was eliminated from the analysis. Therefore, the *Stentor* almost takes the droplet shape caused by the contraction of the trumpet shape. Then, the contracted droplet cell becomes cone shaped and swims away (Figure 2A) or becomes trumpet shaped again (Figure 2C). Additionally, the duration times are 16.2 ± 0.2 s (SD, *n* = 13, 10 cells, Figure 2B) and 40.1 ± 0.51 s (SD, *n* = 10, eight cells, Figure 2D). Figure 2E shows another elongation process from cone to trumpet, and the duration time was 107.9 ± 1.47 s (SD, *n* = 20, 14 cells, Figure 2F). The contractile process from trumpet to droplet is very quick, more than ten thousand times faster than other transitions (Figure 2G). In the contraction process, the two-mode exponential function (Moriyama et al., 1998) fits well [2.1 ± 0.51 ms and 15.6 ± 2.3 ms (SD, *n* = 8, eight cells, Figure 2H)] with the averaged deformation rate rather than the function with a single time scale (details in Supplementary Figure S10).

### 3.3 Behavioral switching from swimming to adhering

**FIGURE 3:**
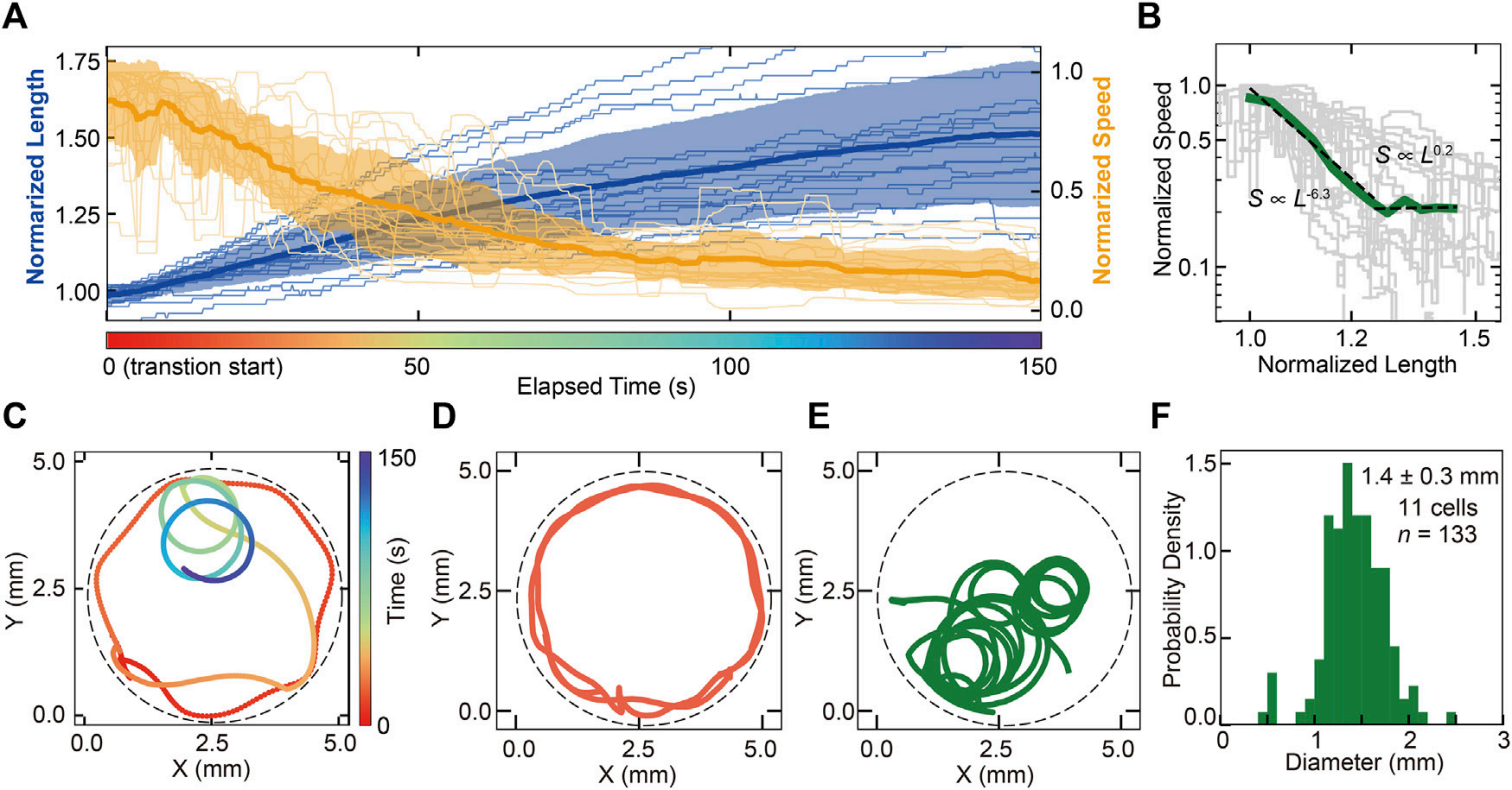
The processes of behavioral switching from swimming (cone shaped) to adhering (trumpet shaped). **(A)** Time series of the cell length (blue line) and swimming speed (orange line) of the cell in the transition from cone to trumpet (Figures 2E,F). Averaged lines are represented as solid bold lines, and individual data are represented as solid thin lines. Standard deviations of values for each cell at a time point are the transparent regions. These values are normalized by Cone = 1. It shows the negative correlation between the cell length and swimming speed. **(B)** The correlation between cell length and speed. The green solid line represents the average speed. Gray lines are individual transition data, and averaged data are fitted by power laws. The characteristic factors of the law are −6.3 in the range of 1–1.25 (normalized length) and 0.2 in the range of 1.25–1.45 (normalized length). **(C)** The trajectory of a cell in the behavioral transition from swimming to adhering. As the cell elongates, the cell becomes locally rotating. The color represents elapsed time from the beginning of the transition. The dashed line represents the wall of the chamber. **(D,E)** The comparison between the swimming trajectories at swimming steady state and steady rotating state. **(F)** The distribution of the swimming pass diameter in the steady rotating state. The diameter was 1.4 ± 0.3 mm (SD, *n* = 133, 11 cells).

In this section, we focus on the transition from cone to trumpet (Figures 2E,F) related to the determination of the adhering location. In the transition, the swimming speed of the cell decreases, accompanied by increasing cell length (Figures 3A,B). The correlation between the length and speed of the cell shows a negative correlation, and two kinds of relationships are observed in the transition by scaling methods (*s*∼*l* ^−6.3^ and *s*∼*l* ^0.2^, Figure 3B). In the behavioral transition, the swimming trajectory changed from straight forward to rotating (Figure 3C, Supplementary Video S1). The trajectories in the swimming and trumpet states are shown in Figures 3D,E. The true diameter of the trajectory in the free-swimming cone state could not be measured because the cone-shaped cell usually swims in a straight trajectory and the swimming is affected by the chamber’s wall due to the restriction of the observation area. The diameter of the rotating trajectory of the trumpet cell (Figure 3E) is distributed at 1.4 ± 0.3 mm (SD, *n* = 133, 11 cells, Figure 3F). The parameters of the beating forms of the membranellar band are different between the cone state and trumpet state (Supplementary Figure S11).

### 3.4 The influence of a structure on behaviors in an arena

**FIGURE 4:**
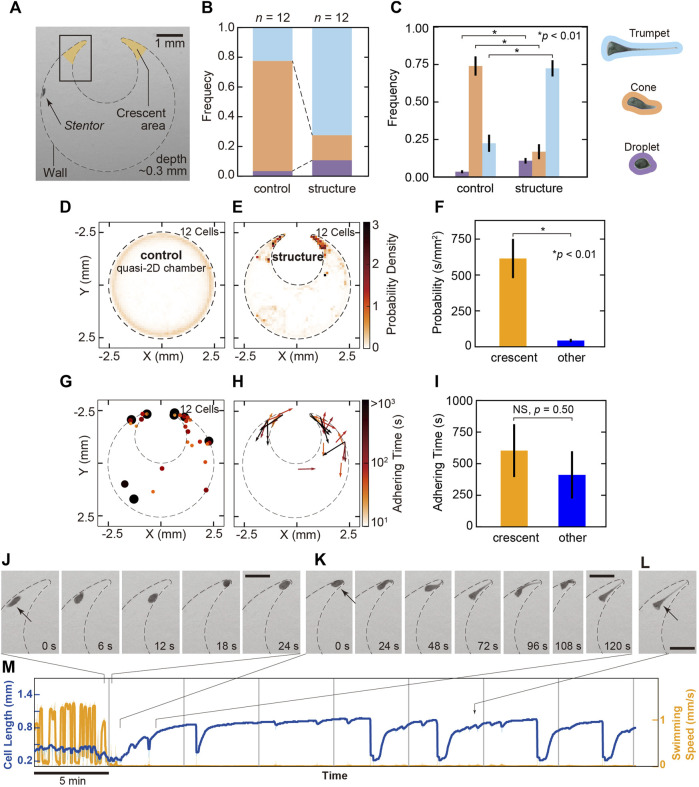
The behavioral change in swimming to adhesion in the crescent areas. **(A)** Image of the geometry of the gel chamber with the structure in the experiments. The dashed line represents the wall of the chamber. Crescent areas are indicated as two orange regions. The depth of the chamber is 0.3 mm. **(B)** The frequency of the cell states in the gel chamber with/without the structure. In the chamber without the structure (control), the cell mainly takes the cone state in 74.1% (orange), and trumpet (light blue) and droplet (purple) states are observed in 22.4% and 3.5%, respectively. In the chamber with the structure, the cell mainly takes a trumpet shape in 72.4% and a droplet shape in 10.7%. Cone-shaped cells are observed in only 16.9% of cells. **(C)** The difference in the probability of three states in the simple chamber and in the chamber with structure. The colors correspond with each state indicated in **(B)**. This bar plot exhibits the probability of cell states in the simple chamber and the chamber with structure. The cone shape was dominant in the simple chambers, but the trumpet type was dominant in the chambers containing structures. The probability of the cell states was tested by Welch’s *t*-test, revealing significant differences between the chamber with/without structure (*p* = 0.0026 in droplet; *p* = 6.3 × 10^−7^ in cone; *p* = 2.0 × 10^−6^ in trumpet). The bars are standard errors (12 cells). **(D,E)** 2D maps of the probability density observed in the cells in each chamber. **(F)** The comparison of presence probability within the crescent areas and other regions. Except for adhering, presence probabilities are 614.9 ± 136.6 s/mm^2^ (SE, *n* = 12, 12 cells) in crescent and 43.4 ± 11.1 s/mm^2^ (SE, *n* = 12, 12 cells) in other regions. The difference is statistically significant in Welch’s *t*-test. Black bars are standard errors. **(G)** The locations and duration times of adhesion in the cell. The color and size of the dots represent the duration of the adhesion. The color bar represents the logarithmic adherence time scale. **(H)** The directions of the cell in the adhesive state. The color and length of the vectors represent the adhering time in one direction. **(I)** The comparison of adhering time prior to the detachment within the crescent areas and other regions. Ten cells adhered in crescent areas (18 events) and 7 cells adhered in other regions (13 events) in observed 12 cells (31 events). Averaged adhering times prior to detachment are 603.0 ± 209.2 s (SE, *n* = 18, 10 cells) in the crescent areas and 411.2 ± 187.1 s (SE, *n* = 13, seven cells) in other regions. Welch’s *t*-test revealed that they did not have a significant difference (*p* = 0.50). Black bars are standard errors. **(J–L)** Typical sequential images of the process from swimming to adhesion in the crescent area [in the rectangle in **(A)**]. **(J)** Before the cell adheres, the cell enters the crescent area. **(K)** Adhesive cells become trumpet-shaped from cone-shaped cells. **(L)** The cell adheres and feeds in the crescent area. The direction of the anterior end directs toward the opposite side to the dead end. All bars are 0.5 mm. **(M)** Time series of the cell length and swimming speed in the process from swimming to adhesion at the crescent area. Images in **(J)**–**(L)** represent the sections in the graph.

In nature, *Stentor* elongates and adheres to a better location for better living conditions. In these processes, the cell obtains some information about the outer environment. To determine where they tend to adhere, we focused on the geometry in the outer swimming environment and observed their behaviors in the quasi-2D gel chambers to encourage cell adhesion with/without the structure (Figure 4A).

The structure in the chamber causes a change in the frequency of the cell states. In the chamber without the structure (control), the cell mainly takes the cone state in 74.1%, and trumpet and droplet states are observed for 22.4% and 3.5%, respectively (Figure 4B). However, in the chamber with the structure, the cell mainly takes a trumpet shape in 72.4% and a droplet shape in 10.7%. Cone-shaped cells were observed in only 16.9% (Figure 4B). In the chamber with the structure, trumpet- and droplet-shaped cells mostly adhere, and swimming cells often collide with the structure. Thus, a distribution of lower swimming speeds is often observed (Supplementary Figure S12A,C). The averaged each state have significant differences in Welch’s *t*-test (*p* = 0.0026 in droplet, *p* = 6.3 × 10^−7^ in cone, *p* = 2.0 × 10^−6^ in trumpet, Figure 4C). The geometry in the outer environment affects the modulation of their states.

The 2D color maps in Figures 4D,E represent the averaged probability densities of the cell’s position in each chamber. In the quasi-2D chamber without a structure, the probability density is uniformly distributed along the wall (Figure 4D). On the other hand, the cell frequently stays in the locations between the wall and the structure, which are termed the crescent areas (in the orange area in Figure 4A) (Figure 4E). Except for adhering, existence frequencies were 614.9 ± 136.6 s/mm^2^ (SE, *n* = 12, 12 cells) in crescent and 43.4 ± 11.1 s/mm^2^ (SE, *n* = 12, 12 cells) in other regions. Welch’s *t*-test was applied to the values, revealing a significant difference between them (*p* = 0.0015, Figure 4F). It shows the increase of the presence in the crescent areas.

Our experiment suggests that the cell tends to stay in and adhere to the crescent areas. Figure 4G displays the positions and duration times of adhesion in 12 cells. All 12 cells adhere to substrate in the chamber with the structure. Though two cells did not adhere to the crescent areas, the others (10 cells) exhibited switching behavior from swimming to adhesion (from cone to trumpet) in crescent areas. In the 10 cells, 9 cells maintained their feeding (trumpet state) with the oral structure directed to the opposite side of the dead end (Figures 4G,H,L). Although the adhering frequency in crescent areas (28.0 events/mm^2^) increases compared to the one in other regions (0.9 events/mm^2^), the difference between adhering times per cell prior to the detachment were not significant in Welch’s *t*-test [603.0 ± 209.2 s (SE, *n* = 18, 10 cells) in crescent areas and 411.2 ± 187.1 s (SE, *n* = 13, seven cells) in other regions; *p* = 0.50; Figure 4I].

Figures 4J–L shows typical sequential images of the adhesion processes in the crescent area (Supplementary Video S2). First, the cell encounters the crescent area and goes back and forth (Figure 4J). Then, the posterior shortly elongates and contacts the wall. The cell sometimes swims backward. Then, the posterior region adheres to the substrate and elongates over 1–2 min (Figure 4K). In this process, we can sometimes observe the bending behavior (sequential images from 96 to 120 s in Figure 4K). Although contraction and elongation between the trumpet and droplet occur, the cell typically remains adherent at the same location for more than 35 min (Figure 4M, Supplementary Figure S9).

Modulation of their states is affected by the structure in an arena. In the crescent areas, the cell not only stays frequently but also switches its behavior and adheres for a long time with its oral structure directed to the opposite side of the dead end.

## 4 Discussion

Protists perform some behavioral modulations according to their surrounding environments. The ciliate *Stentor* exhibits changing shape and motility, and several studies have shown the complexity of these behavioral transitions (Jennings, 1906; Wood, 1969b; Dexter et al., 2019; Trinh et al., 2019). The environmental information around the cell affects the behavioral transitions. Here, we quantitatively evaluated the behaviors of *S. coeruleus* and then measured the characteristic durations of their behavioral transitions. Considering the swimming speed and cell length, the cell states are computationally classified into three states, consistent with the previous descriptive classification of the three states (Tartar, 1961). Among those three states, we revealed that *S. coeruleus* has 4 transition processes accompanied by each characteristic duration time. Previously, the durations time from droplet to trumpet and trumpet to droplet have been estimated by qualitative observations, and our results are consistent with their reports (Wood, 1969a; Kristensen et al., 1974). The contraction process in *S. coeruleus* from trumpet to droplet contributes to the contractile filaments, myoneme (Randall and Jackson, 1958; Huang and Pitelka, 1973). The contraction time in *Vorticella convallaria* (Moriyama et al., 1998), which also uses a similar contractile filament, the spasmoneme (Lynn, 2008) is almost the same as that in *S. coeruleus*. In this paper, we also characterized the duration of the elongation processes from droplet to cone and from cone to trumpet. The elongation processes are associated with the sliding of microtubule ribbons in the km-fibers (Randall and Jackson, 1958; Huang and Pitelka, 1973). In our study, the duration times of each elongation were different. This means that the question of whether all three elongation processes relate to sliding in the km-fibers is still open.

In the transition from cone to trumpet, we found a negative correlation between the cell length *l* and swimming speed *s* with the power laws *s*∼*l* ^−6.3^ and *s*∼*l* ^0.2^ (Figure 3B). Hydrodynamically, in a moving sphere (radius *r*) with the same energy, the swimming speed is proportional to *r* ^−1^ by Stokes law. We found that the exponent between observed length and speed is different from that derived from the stokes law. It is considered that the difference in the relationship between our experiment and the theory is derived from the difference in the ciliary waveforms between the cone state and the trumpet state (Supplementary Figure S11, Supplementary Video S3).

The changes in swimming speed and trajectory in *S. coeruleus* play crucial roles in exploring feeding location. In microorganisms, the modulations induced by taxis-caused chemical substances, light and flows have been well studied experimentally and mathematically (Wan and Jékely, 2021). In *Stentor*, photoresponse (Song et al., 1980a; Song et al., 1980b; Iwatsuki, 1992), external mechanical stimuli (Wood, 1969b) and chemical avoidance responses (Jennings, 1906; Dexter et al., 2019; Trinh et al., 2019) have been reported previously. The behaviors of microorganisms are strongly influenced by extracellular geometries (Kantsler et al., 2013; Kunita et al., 2014; Beppu et al., 2017; Beppu et al.,2021; Ishikawa and Kikuchi, 2018; Nishigami et al., 2018; Ohmura et al., 2018; Ohmura et al., 2021; Ostapenko et al., 2018; Bhattacharjee and Datta, 2019; Bentley et al., 2021; Okuyama et al., 2021; Théry et al., 2021) corresponding to mud, dead leaves or other varieties of sediments in their habitats. In our study, we evaluated the effect of a structure on behavioral transition. In the chamber with the crescent areas, the probability density of the cell’s position at the crescent areas is higher, and the cell becomes more adherent than the cells at other locations. Although the frequency of presence except for adhering had a significant difference, adhering time per cell prior to detachment did not have a significant difference. These statistical analyses indicated that the bias to adhere to the crescent was dominated by an increase in adhesion than a decrease in detachment.

Previously, a directional swimming organism such a *Chlamydomonas* accumulates the corner in the geometrical chamber, and the phenomenon has been described as a theoretical model (Théry et al., 2021). Although the cell accumulates in the corner without changing the behavioral state (Mondal et al., 2021), we revealed that *S. coeruleus*, which is frequently found in a free-swimming form (Reynierse and Walsh, 1967), tended to adhere to the chamber with the structure, and the behavioral change promoted accumulation in the crescent areas. The anterior end of the cell faces the opposite side of the dead end. Hydrodynamically, *S. coeruleus*, which belongs to the puller-like swimmer in the squirmer model, tends to swim away from the wall (Lighthill, 1952; Blake, 1971; Lauga and Powers, 2009). When the posterior region of the cell, which secretes a sticky substance, attaches to a substrate (Andrews, 1945), the cell directs the anterior toward the opposite side of the dead end; this plays a role in positive feeding efficiency.

Moreover, it is known that the *Stentor* causes two feeding vortices at the left and right sides of the oral apparatus on a focal plane (Maupas, 1888). Proximity to the attachment surface or substrate causes vortex flow near the sessile filter feeders, *Vorticella* and *Opercularia,* generating feeding currents around the oral apparatus similar to *Stentor* (Liron and Blake, 1981; Hartmann et al., 2007; Pepper et al., 2010, 2013). The feeding efficiency influenced by the feeding vortex depends on the environmental situations and theoretical definitions. The eddies contribute to decreasing the long-term feeding efficiency defined by the clearance rate (the volume of nutrient water crossed per time) due to recirculation when we consider that there is no nutrient in the flow processed the oral region once (Liron and Blake, 1981; Hartmann et al., 2007; Pepper et al., 2013, 2021). On the other hand, when we modeled that the organisms probabilistically capture prey, the vortex array contributes to the positive efficiency of their feedings because the recirculation allows an increase in feeding chances of starfish larvae (Gilpin et al., 2017). Additionally, vortex flow enhances the mixing and transport of substances, i.e., nutrients and wastes, around the cell (Mondal et al., 2021). Combining our results and these reports, we determined that *S. coeruleus* adheres to narrow areas not only by avoiding being the prey of potential predators but also by capturing prey and mixing nutrients around the cell.

How organisms perceive the narrowness and shape of space is an interesting future topic. Two possibilities are discussed here: 1) the interaction between the flow in the narrow space and the ciliary beating that causes it, 2) the concentration of some chemical secretions (due to suppression of diffusion by narrow space). The *Stentor* swims by moving their cilia to move the water around the body. Ciliary beating is modulated in response to the reaction force cilia receive from water. This modulation is the interaction of the cilia with the surrounding flow of water (Brumley et al., 2015; Wan et al., 2020). In a narrow space, the surrounding flow changes, so it may react to the change in the flow field around the body (Liron and Blake, 1981; Pepper et al., 2010). Sensing a geometric shape may also be possible in principle since the flow field pattern changes with the spatial shape. In other words, it is a mechanism that can be called *hydrodynamic sensing* for spatial shape. On the other hand, the second possibility is *chemical diffusion sensing*. The *Stentor* secretes various chemical substances, such as signal substances and excretory substances (Andrews, 1945, 1946; Miyake et al., 2001). These chemicals move away from the body by diffusion and advection if the space is sufficiently open. However, in a narrow space, these outflows are suppressed, and the concentration becomes high. This change in a chemical concentration may be a cue for the organism to switch the swimming behavior.

The above two effects do not have to be mutually exclusive, and both may work as possible. Alternatively, in a narrow space where the flow tends to stagnate, bacteria and other microorganisms are likely to grow, and it is possible that the *Stentor* will react to it. In any case, the ability of *Stentor* to perceive the narrowness and shape of space simply by moving around in space, in other words, without perceiving the overall shape from a bird’s-eye view, is surprising in terms of the information processing ability of living things.

## Data Availability

The raw data supporting the conclusion of this article will be made available by the authors, without undue reservation.
