# Supplementary material for: Switching of behavioral modes and their modulation by a geometrical cue in the ciliate Stentor coeruleus
Source: Front Cell Dev Biol. 2022 Nov 1;10:1021469. doi: 10.3389/fcell.2022.1021469 (PMC9663998; doi:10.3389/fcell.2022.1021469)
Supplement: Supplementary file 2 [file DataSheet1.pdf]

Supplementary Material

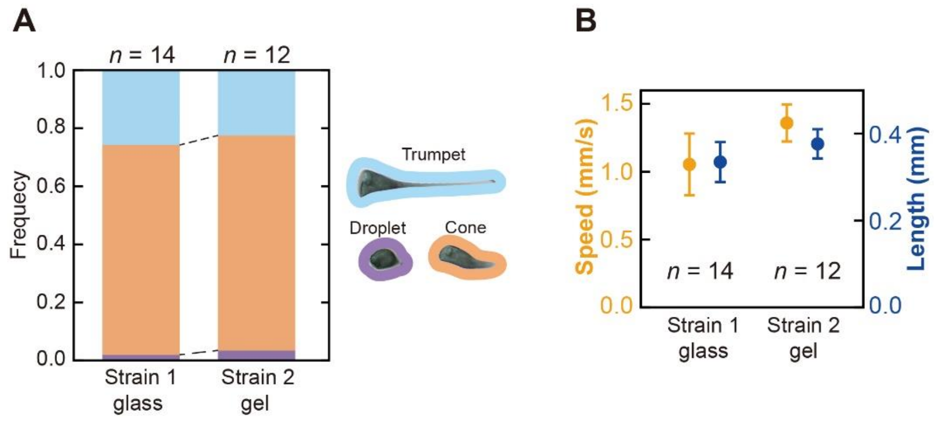

Supplementary Figure 1. The differences between the 2 strains.

(A) The frequencies of the cell states. In strain 1 (obtained from Tomakomai) in a glass chamber, cone-, trumpet- and droplet-shaped cells were observed in 72.3%, 25.7% and 2.0% of cells, respectively. In strain 2 (obtained from Shiribetsu River) in the gel chamber, cone-, trumpet- and droplet-shaped cells were observed in 74.1%, 22.4% and 3.5% of the cells, respectively.

(B) Average cell length and swimming speed in the swimming state. In strain 1 (obtained from Tomakomai) in a glass chamber, the speed and length were 1.05 ± 0.23 mm/s and 0.33 ± 0.046 mm (SD, n = 14), respectively. In strain 2 (obtained from Shiribetsu River) in the gel chamber, the speed and length are 1.35 ± 0.14 mm/s and 0.38 ± 0.034 mm (SD, n = 12), respectively.

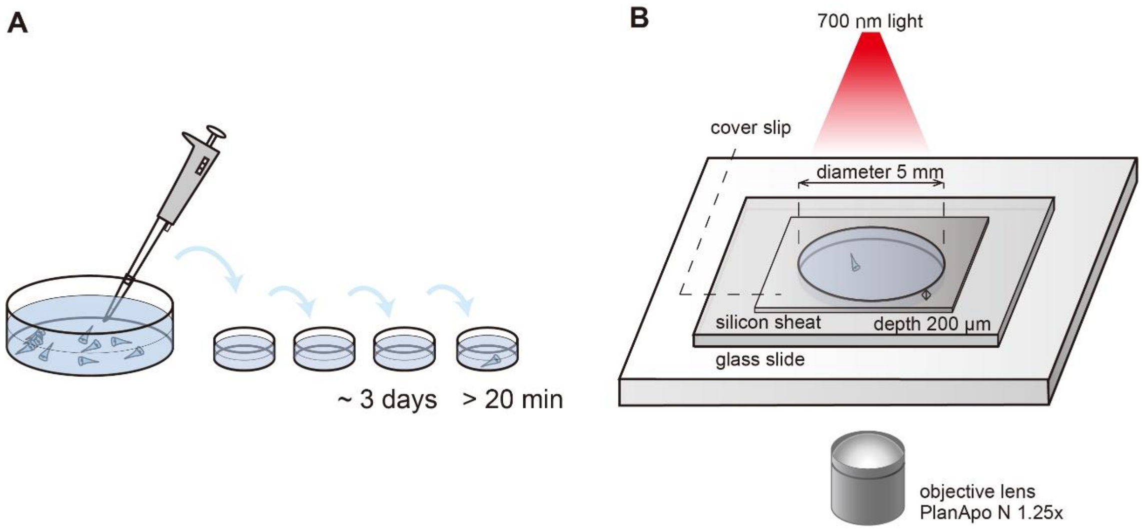

Supplementary Figure 2. Experimental setups.

(A) Before the observations, we washed the cells in fresh pond water twice and starved them for 3 days.

Prior to transferring a cell into an observation chamber, we washed it with pond water again and equilibrated it for over 20 min.

(B) The observation chamber was made by a silicon sheet (0.2 mm depth, 5 mm inner diameter) and covered on a cover slip. Cell behavior was observed by an inverted microscope equipped with the objective PLANPO X1.25 under a bright field using 700 nm light.

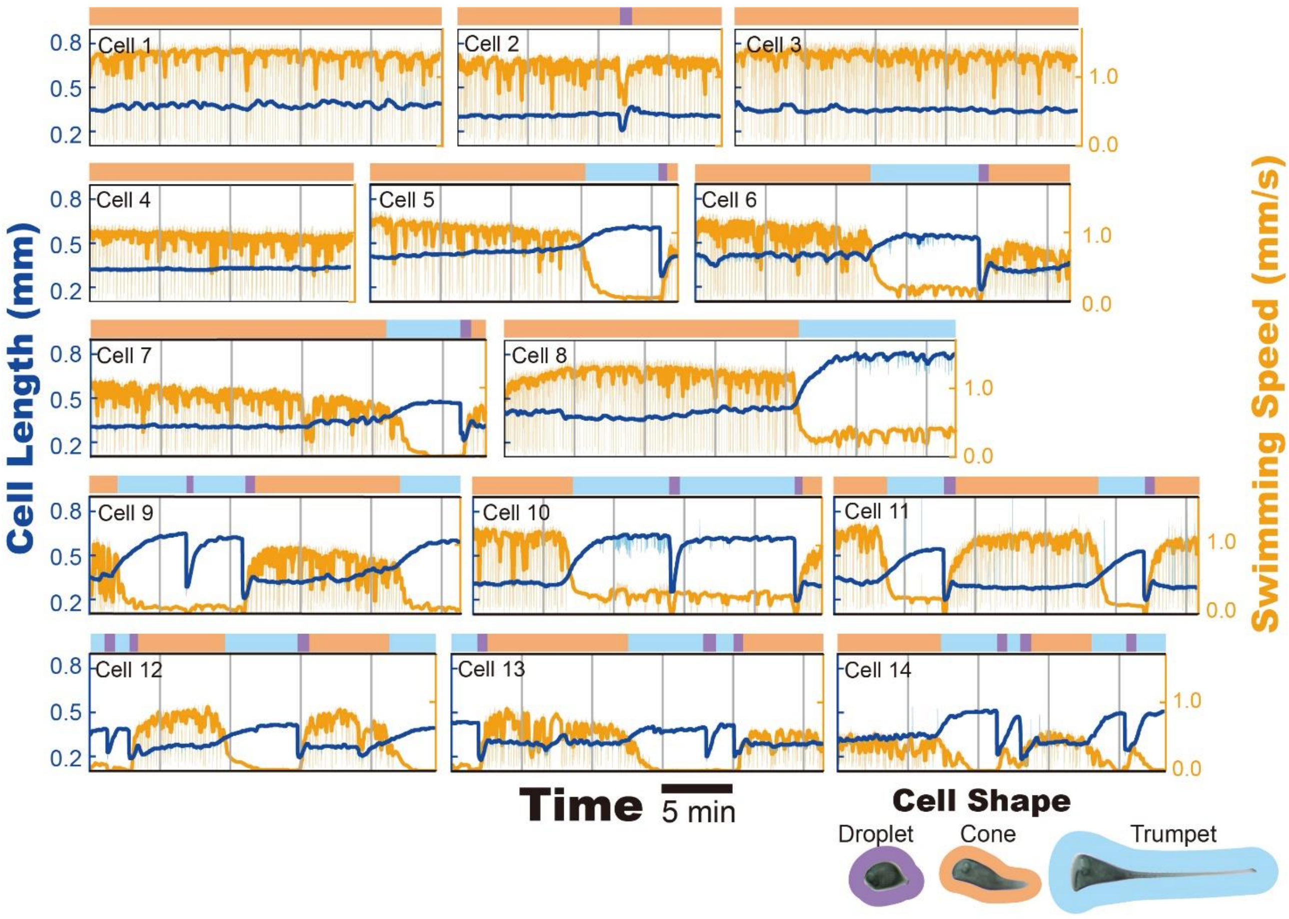

Supplementary Figure 3. The time series of the cell length (blue line) and swimming speed (orange line) in 14 cells.

We used 14 cells to obtain the frequencies of each cell state. We recorded each cell for 24.7 ± 3.4 min (SD, 14 cells, maximum 32.0 min, minimum 18.6 min, total 345.1 min). Some cells take the steady cone state (Cells 1–4), others change their states frequently (Cells 9–14). There is a variation in the frequency of changing the states among those cells (Cells 5–8).

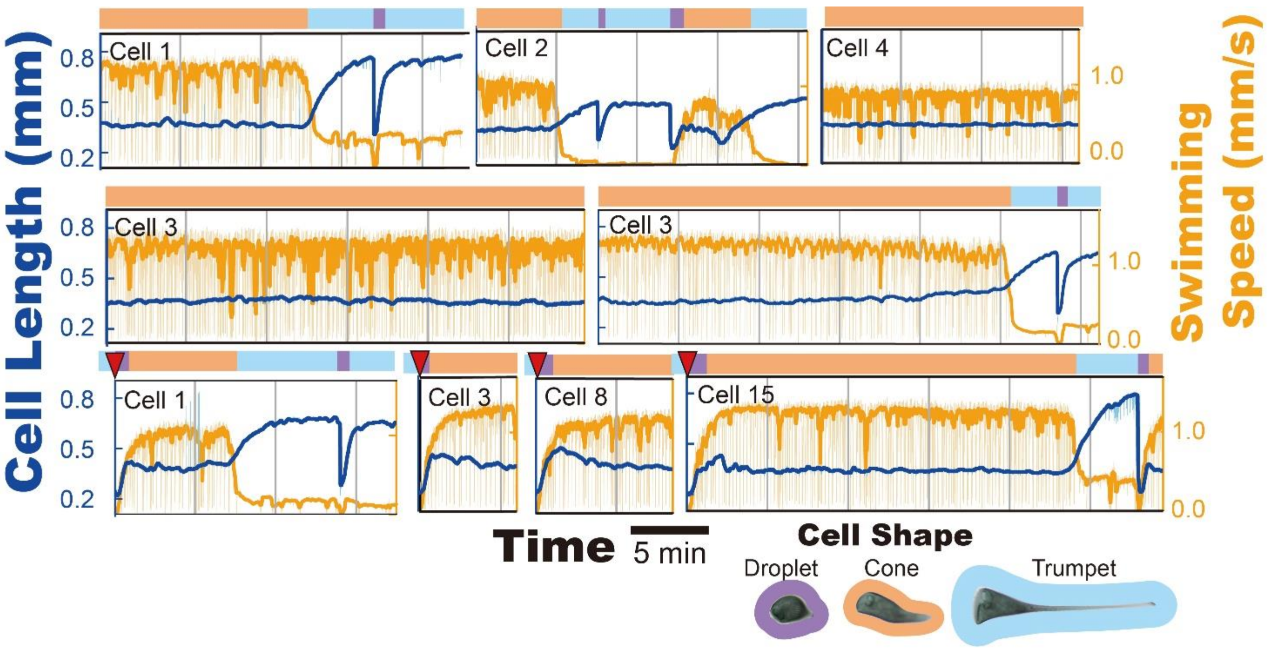

Supplementary Figure 4. Additional data for quantitative measurements of cell behavior.

To measure the duration time of each shape transition and the diameter of the swimming trajectory in the trumpet shape, we recorded the cell behaviors and analyzed the data. Because of the very low frequency at the droplet state, we recorded further behaviors of the cells in the droplet state by adding an external mechanical stimulus (red rectangle symbol) to obtain the transition vector and conduct the computational classification of the cell state. The indices of the cell correspond to those in **Supplementary Figure 3**.

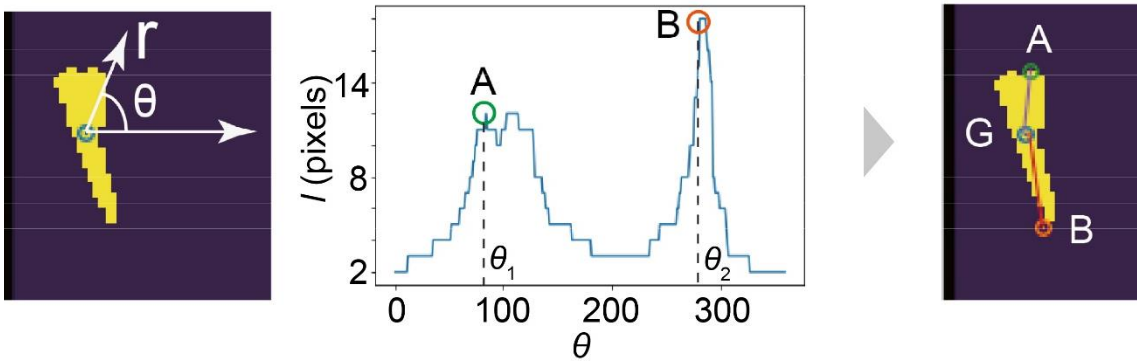

Supplementary Figure 5. Measurement of the cell length.

We obtained the length $l_i(\theta)$ from the center G of the cell to the edge by converting the images to polar coordinates by 2°. The cell length L_i was defined by L_i = AG + BG = $l_i(\theta_1) + l_i(\theta_2)$, where $l_i(\theta_1)$ and $l_i(\theta_2)$ are the top two local maxima of $l_i(\theta)$, and θ_1 and θ_2 denote the anterior and posterior directions, respectively.

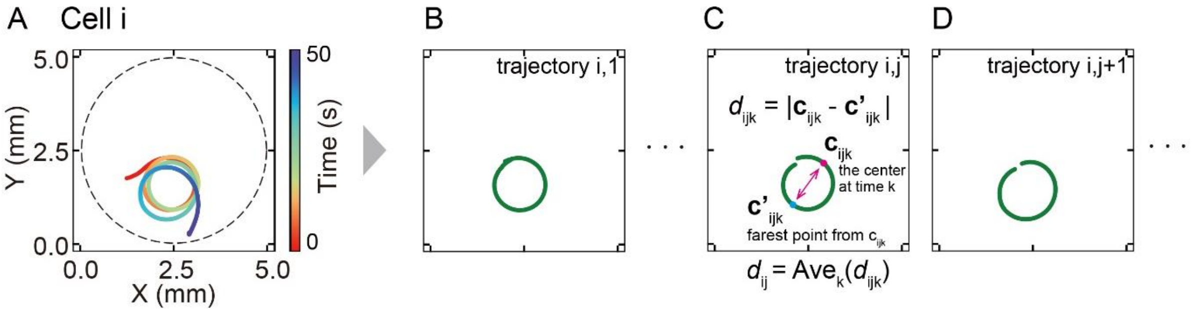

Supplementary Figure 6. Measurement of the diameters of each trajectory.

First, we divided the rotating trajectories of cell i **(A)** into each lap (j-th lap) denoted by "trajectory i, j" **(B–D)**. The diameter of "trajectory i, j", namely, d_{ij}, was calculated by the maximum distance d_{ijk} between the center at the k-th frame and other centers in "trajectory i, j" and averaging d_{ijk} with respect to k **(C)**. The histogram of d_{ij} is shown in **Figure 3F** in the main text.

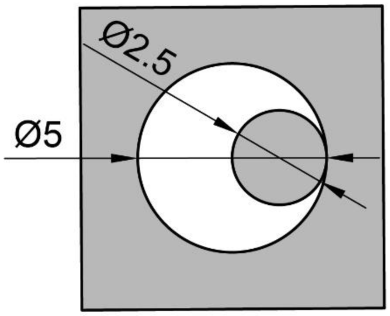

Supplementary Figure 7.

The drawings of the gel chamber containing a disk-like structure (diameter 2.5 mm, 0.3 mm depth) inscribed in the quasi-2D disk chamber (diameter 5 mm, 0.3 mm depth). The observation chambers were obtained by mold casting in two processes: the preparation of prime molds made of polydimethylsiloxane (PDMS) and the formation of a gel chamber by pouring agarose gel into the PDMS prime molds.

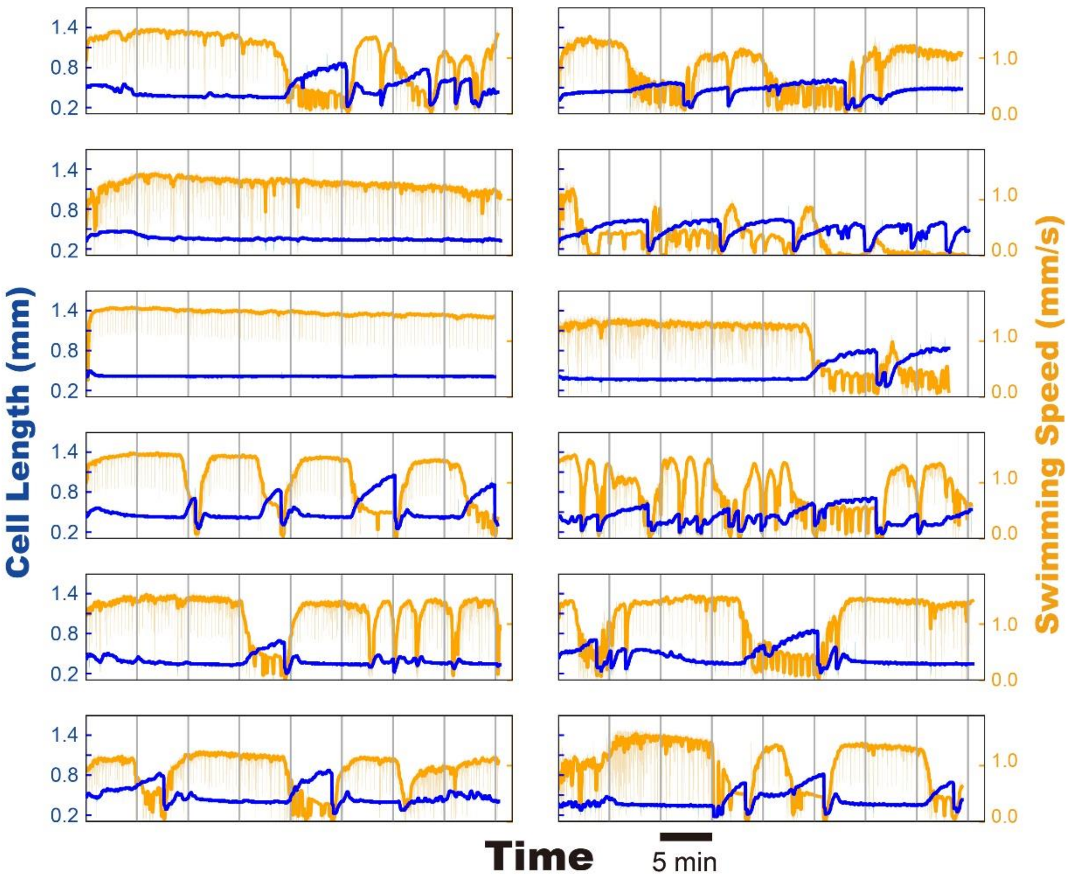

Supplementary Figure 8.

The time series of the cell length (blue) and swimming speed (orange) in the chamber without the crescent areas.

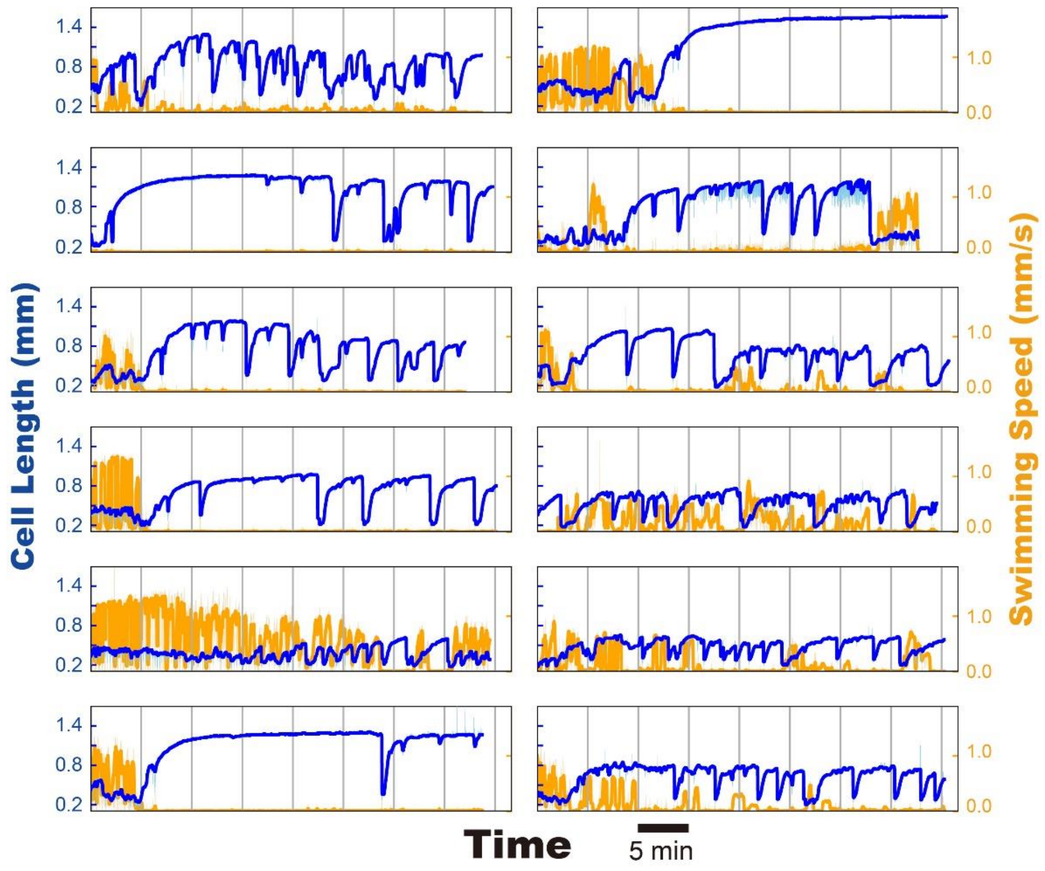

Supplementary Figure 9.

The time series of the cell length (blue) and swimming speed (orange) in the chamber with the crescent areas.

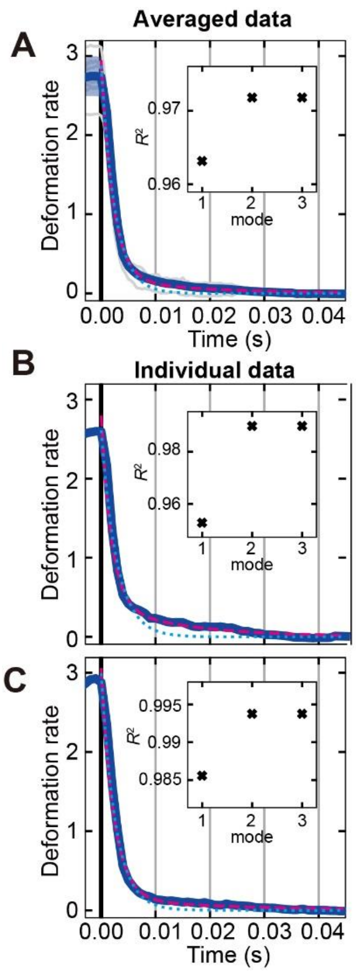

		T_1	T_2	T_3
··········	1 mode	2.52 ms	-	-
- - - - -	2 modes	2.14 ms	15.61 ms	-
not shown	3 modes	2.14 ms	2.14 ms	15.61 ms

Averaged line (n = 8, 8 cells)

		T_1	T_2	T_3
··········	1 mode	2.88 ms	-	-
- - - - -	2 modes	1.90 ms	14.52 ms	-
not shown	3 modes	1.90 ms	1.90 ms	14.52 ms

individual data

		T_1	T_2	T_3
··········	1 mode	2.62 ms	-	-
- - - - -	2 modes	2.29 ms	20.67 ms	-
not shown	3 modes	2.29 ms	2.29 ms	20.67 ms

Individual data

Supplementary Figure 10.

(A) In the contraction process, the two-mode exponential function fits well (2.1 ms and 15.6 ms (n = 8, 8 cells)) to the averaged deformation rate rather than the function with a single time scale. The inset of the graph indicates the coefficient of determination R^2 regarding the numbers of modes.

(B, C) In the two individual contraction processes, the two-mode exponential function also fits well to the deformation rate rather than the function with a single time scale. The inset of the graph indicates the coefficient of determination R^2 regarding the numbers of modes.

The results indicate that individual contraction has two characteristic times in the quick contractile process from trumpet to droplet, but there are not two types of samples.

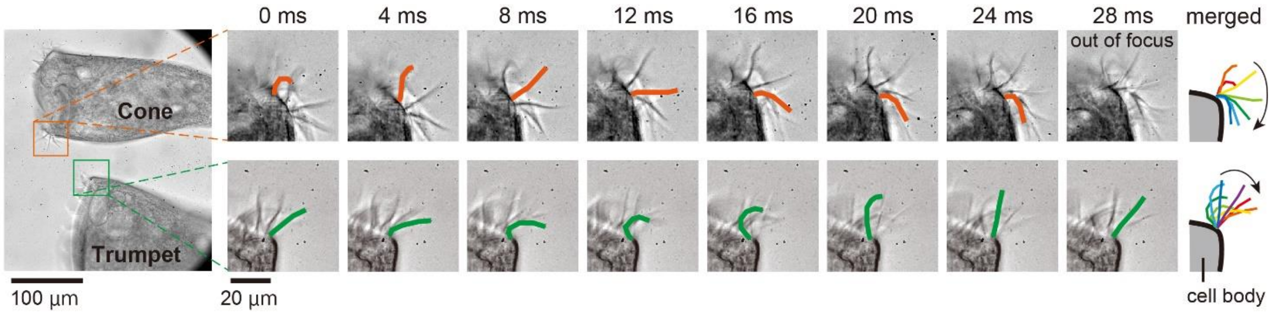

Supplementary Figure 11. The difference in the beating forms of the ciliary band (membranellar band) in *S. coeruleus* in cone and trumpet states.

We used the cells collected in the Shiribetsu River and cultured them by the methods described in Section 3.1. Before observation, we washed the cells with fresh modified Peters' solution two times. After 1–2 h, we transferred the cells into a chamber made with a silicon sheet (5 mm diameter and 0.2 mm depth) and covered the chamber with a cover slip. The beating forms were observed by an inverted microscope IX73 equipped with the objective UPLSAPO60XW (Olympus, Tokyo, Japan) and recorded by using the FASTCAM Mini AX50 at 2,000 fps and an exposure time of 1/2,000 s under a brightness field using weak 700 nm light.

We manually traced one membranellar band every 4 ms. Due to 3D beating, we could not extract the segmentation lines in the recovery strokes in the cone state. The beating frequencies are approximately 15 Hz in the cone state and 30 Hz in the trumpet state, and the angles of the beat are approximately 130 degrees in the cone state and 70 degrees in the trumpet state. The parameters of the beating form of the membranellar band are different. The differences may contribute to the switching motility from a straightforward swimming trajectory to a rotating one.

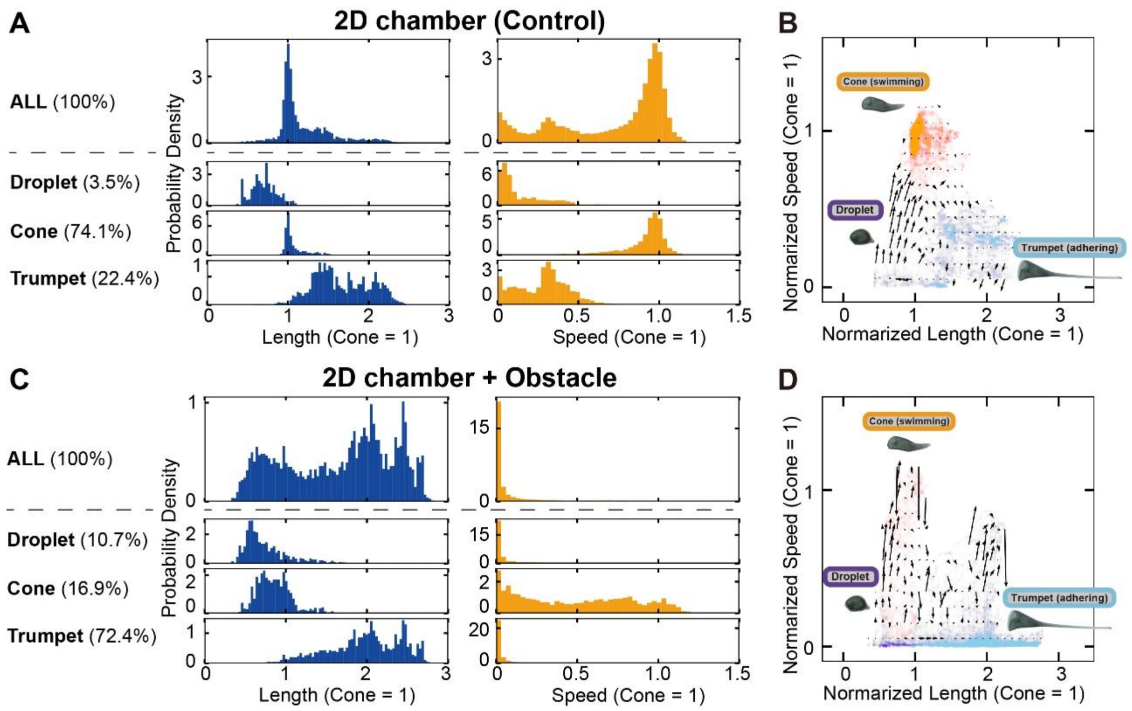

Supplementary Figure 12. The frequencies of the cell states and transition vectors in the quasi-2D gel chamber with/without a structure.

(A) The frequency of the cell states measured by observation time and the distributions of the cell length and speed in each state. These data are derived from the recorded behaviors of 12 cells for approximately 40 min. Each state manually divided by tartar's classification (Tartar 1961) is observed at 3.5% droplets, 74.1% cones and 22.4% trumpets without a structure.

(B, D) The transition vector of the cell. The dots represent the cell states in the 2D 'physical' state field containing the cell length and speed at time points. The dots were manually classified into three by Tartar's descriptive classification of the cell shape (droplet, purple; cone, orange; trumpet; light blue). These values are normalized by cone = 1 (see method in detail).

(C) The frequency measured by observation time of each cell state and the distributions of the cell length and speed in each state. These data are derived from the recorded behaviors of 12 cells for approximately 40 min. Each state manually divided by tartar's classification is observed at 10.7% droplets, 16.9% cones and 72.4% trumpets with a structure.

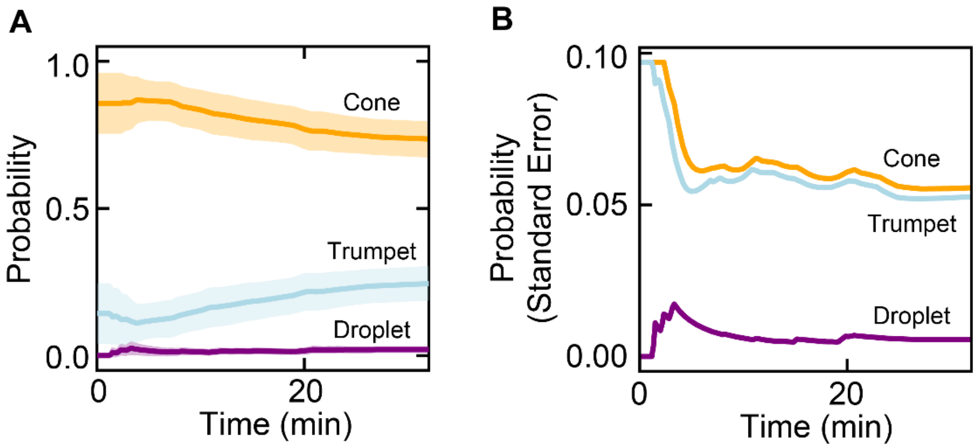

Supplementary Figure 13 | Time series of averaged probabilities and standard errors of each state from the onset of the record.

(A) Solid lines indicate the average probabilities of cell state for the sampling period, and the transparent areas indicate the standard errors of 14 cells. **(B)** The standard errors for the sampling period. The line colors mean the cellular states in **(A)**. These standard errors are almost steady after 5 min.

Supplementary Table 1. The cutting parameters of the preparation of the PDMS chamber. The value in parentheses is the parameter in finish cutting.

	MHRH230	2CEM-G-2.5D-2
Diameter (mm)	0.2	2
Z-axis feed rate (mm/s)	0.3 (0.3)	2.2 (2.2)
Axial depth of cut (mm)	0.01	0.4
X-Y plane feed rate (mm/s)	0.3 (0.3)	2.6 (2.6)
Pass interval (radial depth of cut) (mm)	0.1 (0.1)	0.1 (0.01)
Spindle speed (rpm)	8000	8000

Supplementary movie 1

The switching behavior from cone to trumpet. The movie corresponds to **Figure 3C**.

Supplementary movie 2

The behavior from swimming to adhering at crescent area. The movie corresponds to **Figures 4J** (from 0 s to 24 s) and **4K** (from 70 s to 190 s).

Supplementary movie 3

The movie corresponds to **Supplementary Figure 11**.
